# Solution structure of the YTH domain in complex with N6-methyladenosine RNA: a reader of methylated RNA

**DOI:** 10.1093/nar/gku1116

**Published:** 2014-11-11

**Authors:** Dominik Theler, Cyril Dominguez, Markus Blatter, Julien Boudet, Frédéric H.-T. Allain

**Affiliations:** Institute of Molecular Biology and Biophysics, Eidgenössische Technische Hochschule (ETH) Zurich, 8093 Zurich, Switzerland

## Abstract

N^6^A methylation is the most abundant RNA modification occurring within messenger RNA. Impairment of methylase or demethylase functions are associated with severe phenotypes and diseases in several organisms. Beside writer and eraser enzymes of this dynamic RNA epigenetic modification, reader proteins that recognize this modification are involved in numerous cellular processes. Although the precise characterization of these reader proteins remains unknown, preliminary data showed that most potential reader proteins contained a conserved YT521-B homology (YTH) domain. Here we define the YTH domain of rat YT521-B as a N^6^-methylated adenosine reader domain and report its solution structure in complex with a N^6^-methylated RNA. The structure reveals a binding preference for NGANNN RNA hexamer and a deep hydrophobic cleft for m^6^A recognition. These findings establish a molecular function for YTH domains as m^6^A reader domains and should guide further studies into the biological functions of YTH-containing proteins in m^6^A recognition.

## INTRODUCTION

Methylation of adenine at the N6 position (m^6^A) is considered the most abundant messenger RNA modification in eukaryotes besides the 5′ cap structure (1,2). Functional impairment of methylase function leads to severe phenotypes in a number of organisms such as cell death and developmental arrest (2,3). Genetic alterations in one known demethylase gene (FTO) were associated in humans with increased body mass (4) and higher propensity for cancer (5,6). In recent years several thousand methylation sites have been identified in eukaryotic transcriptomes by the use of next-generation sequencing-based approaches (7–13). Quite consistently, m^6^A are embedded in a consensus sequence in the form 5′ R-R-m^6^A-C 3′ (where R are purines). Two studies used RNA immunoprecipitation and mass spectrometry to identify proteins binding selectively to the m^6^A-containing RNA sequences (7,11). Two out of three top confidence category proteins enriched in the pull-downs with the methylated RNA from HepG2 cell lysates contained one YTH domain (YTHDF2, YTHDF3) (7). The top candidate from meiotic yeast lysates was the YTH domain containing protein MRB1 (11). Full-length proteins YTHDF1, which also contains a YTH domain, YTHDF2 and YTHDF3 were later shown by gel shifts to have increased affinities for the methylated compared to the non-methylated form of the same RNA target sequence (12). This suggested that YTH-containing proteins, whose functions are generally unknown, could act as m^6^A readers.

**Figure 1.**
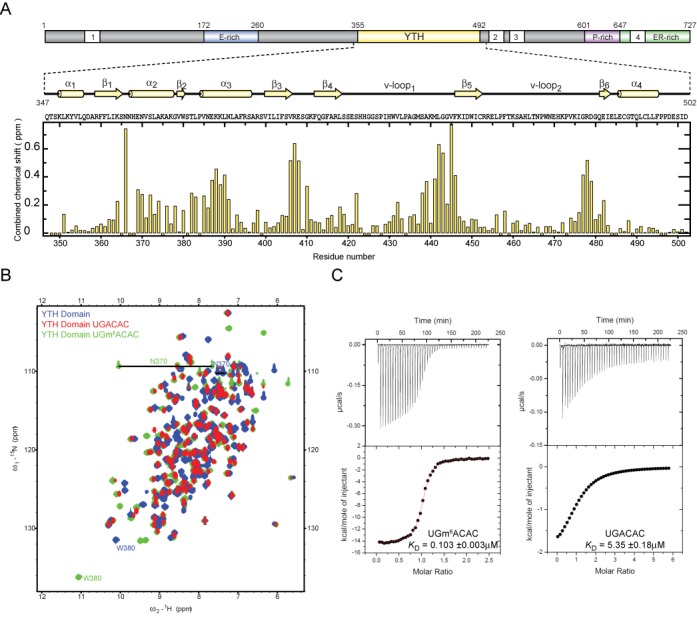
The YTH domain has an increased affinity for m^6^A-containing RNA. (**A**) Top: schematic depiction of the domain organization of *R. Norvegicus* YT521-B. Nuclear localization signals are represented as white boxes numbered 1–4. E-rich, P-rich and ER-rich stand for sequence stretches enriched in glutamate, proline, glutamate and arginine amino acids, respectively (17). Secondary structure elements based on the presented structure are shown. Bottom: combined chemical shift mapping of the *R. Norvegicus* YT521-B YTH domain ^1^H-^15^N backbone resonances upon 1:1 complex formation with 5′-UGm^6^ACAC-3′ plotted against the sequence of the used construct. Perturbations were calculated using the formula: Δδ = [(δHN)^2^ + (δN/6.51)^2^]^1/2^. Proline and residues, which could not be assigned in both states, are represented with negative bars. (**B**) Overlay of ^1^H-^15^N HSQC spectra of the YTH domain of YT521-B (blue), the domain in a 1:1 complex with 5′-UGACAC-3′ (red) and 5′-UGm^6^ACAC-3′ (green). For clarity folded arginine and lysine side-chain resonances were omitted from the overlay. Side-chain resonances of N370 and W380 displaying large chemical-shift perturbations are labeled. (**C**) Isothermal titration calorimetry data of RNA binding to the YTH domain. Left and right calorimetric titration profiles correspond to the 5′-UGm^6^ACAC-3′ and 5′-UGACAC-3′ RNA being injected into the YTH protein, respectively. The upper panels show the raw calorimetric data. The bottom plots are integrated heats as a function of the RNA/YTH molar ratio. Black dots indicate the experimental data. The best fit (depicted by a red line) was obtained from a non-linear least-squares method using a one-site binding model. Heats of dilution have been obtained from independent titration experiments. Both reactions are exothermic.

The first protein containing a YTH domain, which was functionally characterized, is the *Rattus Norvegicus* protein YT521-B (alternative name YTHDC1) (Figure 1A), which was identified in two yeast two hybrid studies aimed at identifying novel alternative splicing regulators using the SR-like protein Tra2β as a bait (14,15). The protein was shown to be able to influence alternative splicing but lacked a previously known RNA-binding domain. Sequence alignment searches identified a conserved domain, which was termed YTH domain for YT521-B homology domain (16). Subsequently, we have shown that the YTH domain of YT521-B was indeed a RNA-binding domain with a very degenerate sequence-specificity (17). A more precisely defined binding sequence containing a triple A motif was later identified by biochemical and bioinformatics approaches for the *Schizosaccharomyces pombe* YTH-containing protein MMI1 (18,19).

Here we show that the YTH domain of YT521-B binds sequence-specifically a GA-containing sequence with a 50-fold affinity increase when the adenine is N6 methylated. We determined the solution structure of the complex which provides the structural basis of the specific m^6^A recognition by the YTH domain. On the basis of the structure, we rationalize why more generally YTH domains act as reader domains for m^6^A.

## MATERIALS AND METHODS

### Protein and RNA preparation

Residues 347–502 of the *R. Norvegicus* YT521-B protein were cloned into the pTYB11 vector (New England Biolabs). The used cloning scheme results after intein cleavage in a protein without any vector-derived residues. *Escherichia Coli* BL21 (DE3) codon plus (RIL) cells were grown in M9 minimal medium containing 1 g/l ^15^NH_4_Cl and 4 g/l unlabeled glucose (^15^N-labeling) or 2 g/l ^13^C-labeled glucose (^13^C^15^N labeling). Protein expression was induced at a cell density of *A*_600_ ≈ 0.6 by addition of 1mM isopropyl-β-D-thiogalactopyranoside. After induction the temperature was reduced from 37°C to 18°C. After overnight expression cells were harvested, resuspended and lysed by cell cracking. Protein purification on chitin columns and intein-mediated cleavage was performed according to the manufacturer's instruction. For cell lysis and intein purification a buffer containing 20 mM Na_2_HPO_4_, 0.5 M NaCl at pH 8 was used. For intermediate washing steps, a buffer containing 1 M NaCl instead of 0.5 M NaCl was used. For cleavage the lysis and intein purification buffer contained in addition 50 mM DTT. After the elution of the YTH domain from the chitin column, the eluate was concentrated with 5 kDa cutoff centricons (Vivaspin) and purified with size exclusion chromatography (SEC) using a Superdex 75 column (GE Healthcare) in a buffer containing 25 mM NaH_2_PO_4_, 25 mM NaCl and 10 mM β-Mercaptoethanol at pH 7 (pH adjusted with HCl) with the addition of 200 units of SUPERase In RNase inhibitor (Ambion) prior to loading of the sample on the SEC column. Fractions containing the YTH domain were pooled and concentrated for further experiments. RNAs were deprotected according to the manufacturer's instructions (Thermo Fisher), lyophilized and resuspended in the size exclusion buffer.

### NMR measurements

Protein RNA titrations were measured with a protein concentration of 0.2 mM and at a temperature of 20°C. Measurements for the structure calculation of the YTH in complex with 5′-UGm^6^ACAC-3′ were performed at a concentration of 0.8 mM at 30°C. Nuclear magnetic resonance (NMR) measurements were carried out in size exclusion buffer either containing 90% H_2_O/10% D_2_O or 100% D_2_O. Spectra were acquired on Bruker AVIII 500 MHz, AVIII 600 MHz, AVIII 700 MHz and AVIII HD 900 MHz spectrometers equipped with cryoprobes. Data acquisition and processing was carried out with Topspin3 (Bruker). NMR data were analyzed with Sparky 3.114 (Goddard T.D. and Kneller D.G., SPARKY 3, University of California, San Francisco).

For protein resonance assignment the following spectra were used: 2D ^1^H-^15^N HSQC, 2D ^1^H-^13^C_aliphatic_ HSQC, 2D ^1^H-^13^C_aromatic_ HSQC, 3D HNCO, 3D HNCA, 3D HNCACB, 3D CBCACONH, 3D ^13^C-^15^N-^1^H HCC(CO)NH-TOCSY, 3D ^1^H-^15^N-^1^H HCC(CO)NH-TOCSY, 3D-NOESY ^1^H-^15^N HSQC (*τ*_m_ = 150 ms), 3D NOESY ^1^H-^13^C_aliphatic_ HSQC (*τ*_m_ = 150 ms), 3D NOESY ^1^H-^13^C_aromatic_ HSQC (*τ*_m_ = 150 ms). All were collected in buffer containing 90% H_2_O/10% D_2_O. For observation of slowly exchanging amide protons a 2D ^1^H-^15^N HSQC in 100% D_2_O was recorded. For RNA resonance assignment the following spectra were used: 2D ^1^H-^1^H NOESY (*τ*_m_ = 150 ms), 2D ^1^H-^1^H TOCSY (*τ*_m_ = 60 ms), 2D F1-filtered F2-filtered ^1^H-^1^H NOESY (*τ*_m_ = 150 ms) (20), natural abundance 2D ^1^H-^13^C_aliphatic_ HSQC and 2D ^1^H-^13^C_aromatic_ HSQC. All were recorded in buffer containing 100% D_2_O. For intermolecular Nuclear Overhauser Effect (NOE) peak assignment a 3D ^13^C F1-edited F3-filtered NOESY ^1^H-^13^C HSQC (*τ*_m_ = 100 ms) collected in 100% D_2_O and a 2D F2 filtered ^1^H-^1^H NOESY (*τ*_m_ = 150 ms) recorded in 90% H_2_O/10% D_2_O were used (20).

### Structure calculation and refinement

Peak picking and initial NOE assignment were carried out with the ATNOS CANDID package (21,22). The resulting peak lists of cycle 7 were used as input for the NOE-assign module of CYANA 3.95 (23). From the resulting upper distance limits lists, distances with a quality factor less than 0.4 were removed. Peak lists were further manually refined. Structure calculations were carried out with CYANA 3.95 using a library containing an entry for m^6^A. Restraints for backbone dihedral angles based on chemical shifts were obtained using the program Talos+ (24). Hydrogen bond restraints were based on protected amides in D_2_O and analysis of initial structures. Intra RNA and intermolecular NOEs were manually assigned and calibrated to be used as input for structure calculation. The structure was refined in the rna.ff12SB force field (25) using the sander module of AMBER12 (26) using a protocol, in which the structures are first minimized and then refined using a simulated annealing protocol with 30 000 steps. Of the 250 structures calculated in CYANA the 50 structures with the lowest target function were refined in AMBER. For the final ensemble the 20 violation energy best structures out of the 30 structures with the lowest AMBER energy were selected. Force field parameters for m^6^A have been obtained from http://ozone3.chem.wayne.edu/ (27). The Ramachandran plot analysis was performed by the program CYANA, which uses the definitions of the program PROCHECK (28).

Figures were generated using MOLMOL (29) and PyMOL (www.pymol.org, Schrödinger, LLC). The electrostatic surface potential was generated with the PyMOL APBS Tools plugin using PDB2PQR (30) and APBS (31).

### Modified scaffold-independent approach

In contrast to the original scaffold-independent approach (32) in the modified approach used here, the nucleotide with the highest score for a position is kept constant for assessment of subsequent positions. Positions of the hexanucleotide were assayed in the order 4, 3, 2, 6 and 1 (Supplementary Figure S1A). Titrations were performed in a buffer containing 50 mM NaH_2_PO_4_, 50 mM NaCl and 10 mM β-Mercaptoethanol at pH 7. Titrations were monitored with 2D ^1^H-^15^N HSQC spectra acquired at 20°C. RNA was titrated to the protein up to a protein:RNA concentration ratio of 1:1.25, except for position 2, for which the ratio was 1:0.6. Combined chemical shift perturbations were calculated using the formula Δδ = [(δHN)^2^ + (δN/6.51)^2^]^1/2^. Peaks in intermediate exchange, which could not be followed over the course of the titration, were assigned a perturbation value of 0.3 ppm. Perturbations for each oligonucleotide assayed were summed up and for each position normalized to the one with the largest perturbation sum.

### Isothermal titration calorimetry measurements

Affinity measurements by isothermal titration calorimetry (ITC) were performed at 20°C using a VP-ITC calorimeter (MicroCal) in identical buffer conditions as described previously for the SEC step. Thermodynamic parameters (*K*, Δ*H*, Δ*S* and *N*) with respective errors (Supplementary Figure S1B) were determined based on χ^2^ minimized fit of the experimental data to a single-site binding model as implemented in the Origin software version 7 (Origin Lab) provided with the VP-ITC instrument. For the binding of 5′-UGACAC-3′, 410 μM of RNA has been injected into 14 μM of YTH. For the interaction with 5′-UGm^6^ACAC-3′, we used 125 μM and 10 μM of RNA and protein concentrations, respectively. The isothermal titration calorimetry (ITC) experiments were set to deliver 45 (6 μl) injections at 300 s intervals. Stirring speed and reference power were 307 rpm and 10 μcal/s, respectively. For each control, heats of dilution were not significant (< 0.015 kcal mol^−1^).

### Alignment and homology models of the YTH domains

Sequences of selected YTH-containing proteins were downloaded from UniProt www.uniprot.org and the YTH containing segments aligned using Jalview (33).

Homology modeling was carried out with the MPI bioinformatics toolkit (34) and its installation of Modeller (35) using PDB entry 2YUD as a template. The experimental structure and the homology models were aligned with the PyMol built-in version of CEAlign (36,37).

## RESULTS

### The YT521-B YTH domain binds specifically GA-containing RNA and m^6^A modification increases its affinity

Although the sequence determined by systematic evolution of ligands by exponential enrichment (SELEX) for YT521-B allowed us to map the RNA-binding surface of the YTH domain, the fact that many protein resonances were absent in the spectra of the complex prevented us to determine its structure (17). We therefore searched for a better binding sequence using a variation of the scaffold-independent analysis method for this protein–RNA complex (32). Our iterative approach yielded a binding preference for the 5′-NGANNN-3′ hexamer motif (Supplementary Figure S1A). Based on this motif further experiments were conducted with the sequence 5′-UGACAC-3′. NMR spectra of the YTH domain of YT521 bound to 5′-UGACAC-3′ displayed a larger number of peaks with sharper resonance linewidth as compared to the SELEX-derived sequence. Indeed, most of the protein resonances in the bound form could be assigned (Figure 1A and B). Interestingly, the presence of the triplet GAC in this motif was similar to the methylation consensus G>A-m^6^A-C identified previously (7,10–11). This prompted us to investigate whether the YTH domain of YT521-B could bind the same sequence with m^6^A in the third position with better affinity.

The NMR titrations of the YTH domain with the hexanucleotide 5′-UGACAC-3′ containing m^6^A_3_ showed that the complex formation shifted from a fast/intermediate to a slow exchange regime clearly indicating an increase in binding affinity mediated by the m^6^A modification (Figure 1B and Supplementary Figure S1C). Subsequent affinity measurements by ITC revealed a 50-fold increase in affinity between the RNA containing m^6^A and the unmethylated adenine in position 3 (Figure 1C and Supplementary Figure S1B). Mapping of the perturbed resonances on the YTH domain revealed that the same RNA-binding surface is used whichever RNA is bound (17) and only the magnitude of the chemical shift perturbations is affected by the methylation. This indicates that the YTH domain binds the unmethylated and the methylated RNA in a similar manner.

### The structure of the YT521B YTH domain in complex with 5′-UGm^6^ACAC-3′ reveals a large binding interface and the directionality of the bound RNA

**Figure 2.**
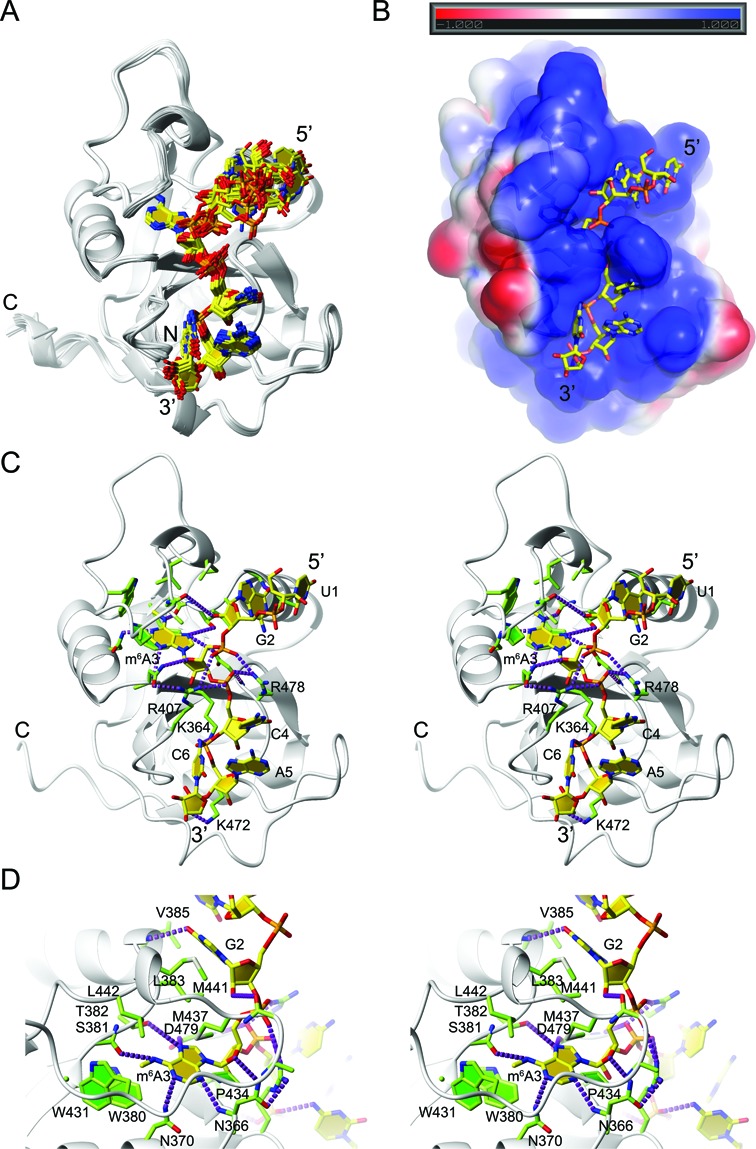
Structure of the YTH domain in complex with 5′-UGm^6^ACAC-3′. (**A**) Ensemble of the 20 selected structures superimposed on the structured residues (Table 1). The YTH domain is displayed as a gray ribbon and the RNA in stick representation of the heavy atoms, carbon (yellow), nitrogen (blue), oxygen (red) and phosphate (orange). (**B**) Electrostatic potential plotted on the surface of the solvent-accessible surface of the YTH domain. RNA is colored as in (A). The surface is displayed partially transparent to visualize m^6^A_3_, which is buried in the hydrophobic core. The ±1 kT/e electrostatic potential is shown with the respective color gradient depicted above the structure with red denoting a negative and blue a positive potential. (**C**) Stereo view of a representative structure of the complex. Depiction as in (A) with the exception of protein carbon atoms shown in green and H-bonds depicted as purple dashed lines. (**D**) Stereo view of the G_2_ and m^6^A_3_ binding pocket. Same depiction as in (A) and (C).

We next determined the structure of the YTH domain of YT521-B (residues 347–502 of the full-length protein, Figure 1A) in complex with 5′-UGm^6^ACAC-3′ RNA using solution state NMR spectroscopy (Figure 2 and Table 1). Using 214 intermolecular NOE-derived distance restraints including 30 to the N6 methyl (Supplementary Figure S2A) a precise ensemble of conformers was obtained (Figure 2A and Table 1). The protein adopts a typical YTH fold, very similar to the YTH structure in its free form (PDB ID 2YUD, Supplementary Figure S2B). The core of the domain is composed of a six stranded β-sheet, which is surrounded by three α-helices (Figure 2A). The RNA adopts an extended conformation and is positioned over the positively charged surface of the YTH domain (Figure 2B). The first two nucleotides U_1_ and G_2_ form a stacking interaction, m^6^A_3_ is looped out with its base buried into the protein (Figure 2B), and the last three residues interact with the protein via their phosphate backbone, with C_4_ and A_5_ stacking together (Figure 2C). G_2_ and m^6^A_3_ are sequence-specifically recognized, while U_1_, C_4_, A_5_ and C_6_ are all in contact with the YTH domain but the structure does not reveal any sequence-specific recognition for these four bases. Hydrophobic contacts to the sugar and base moieties as well as salt bridges to the phosphate oxygens of the RNA backbone provide favorable binding energy to the six-nucleotide RNA (Figure 2C).

**Table 1.** Structural statistics of the YT521-B YTH domain in complex with 5′-UGm^6^ACAC-3'

NMR restraints	
Distance restraints	4760
Protein intramolecular	4497
intraresidual	915
sequential (|*i* − *j* | =1 )	1008
medium range (1 < |*i* − *j* | < 5)	895
long range (|*i* − *j* | ≥ 5)	1644
hydrogen bonds^a^	35
RNA intramolecular	49
intraresidual	30
sequential (|*i* − *j* | =1)	19
Complex intermolecular	214
Torsion angles^b^	241
Protein backbone	234
RNA sugar pucker (DELTA)	6
RNA base conformation (CHI; syn)	1

Energy statistics^c^	
Average distance constraint violations	
0.1–0.2 Å	65.2 ± 4.7
0.2–0.3 Å	6.1 ± 2.3
0.3–0.4 Å	0.6 ± 0.7
> 0.4 Å	0.1 ± 0.3
Maximal (Å)	0.32 ± 0.06
Average angle constraint violations	
< 5°	29.3 ± 2.7
> 5°	0.0 ± 0.0
Maximal (°)	0.51 ± 0.07
Mean AMBER constraint violation energy (kcal mol^−1^)	53.2 ± 2.7
Distance (kcal mol^−1^)	52.6 ± 2.7
Torsion (kcal mol^−1^)	0.6 ± 0.1
Mean AMBER energy (kcal mol^−1^)	−5040.4 ± 11.4
Mean deviation from ideal covalent geometry	
Bond length (Å)	0.0036 ± 0.0000
Bond angle (°)	1.706 ± 0.007

Ramachandran plot statistics^c,d,e^	
Residues in most favored regions (%)	91.2 ± 0.7
Residues in additionally allowed regions (%)	8.8 ± 0.7
Residues in generously allowed regions (%)	0.0 ± 0.0
Residues in disallowed regions (%)	0.0 ± 0.0

RMSD to mean structure statistics^c,d^	
Protein	
Backbone atoms	0.21 ± 0.04
Heavy atoms	0.51 ± 0.05
RNA	
Backbone atoms	0.59 ± 0.31
Heavy atoms	0.64 ± 0.26
All molecules	
Backbone atoms	0.29 ± 0.08
Heavy atoms	0.54 ± 0.06

^a^H-bond constraints were identified from slow exchanging amide protons in D_2_O.

^b^Protein backbone angles determined by the program TALOS+ and sugar pucker angles based on coupling efficiency in homonuclear TOCSY.

^c^Statistics computed for the deposited bundle of 20 violation energy best structure selected out of 30 amber energy best.

^d^Based on structured residue range as defined by user: protein: 351–498, chain ID: A (sequence range: 347–502) RNA : 1–6, chain ID: B (sequence range: 1–6).

^e^Ramachandran plot as defined by the program PROCHECK.

### The YTH domain possesses a buried binding pocket that accommodates the N6-methyl adenine

The N6-methylated adenine adopts an *anti* conformation and its ribose moiety a C2′ *endo* conformation. The YTH-binding pocket is specific for an adenine base making four intermolecular hydrogen bonds: N7 to Thr 382 hydroxyl and Asp 479 sidechain, N6 to Ser 381 carbonyl oxygen, N1 to Asn 370 side-chain amide and N3 to Asn 366 main-chain amide (Figure 2D). The N6-methyl group of adenine 3 is accommodated in a hydrophobic binding pocket involving the side chains of Trp 380, Trp 431 and Leu 442 (Figure 2D). In addition, the adenine base position is stabilized by hydrophobic contacts involving Pro 434, Met 437, Met 441 and Leu 383 (Figure 2D). Not only are all the functional groups of m^6^A perfectly recognized by the YTH domain but this nucleotide is also totally buried within the protein core rendering it inaccessible to the solvent (Figure 2B).

### Recognition of guanine 2

Guanine 2 adopts a *syn* conformation which is stabilized by contacts of its H1′ and H8 with Leu 383 and Met 441 (Figure 2D). Furthermore, the O6 carbonyl of G2 is hydrogen-bonded with the amide proton of Val 385 (Figure 2D). These contacts are sufficient to discriminate a guanine over any of the four nucleotides in this position. This is consistent with the preference for a guanine in position 2 during our optimization of the best binding sequence (Supplementary Figure S1A).

### A possible common mode of binding for all YTH domains

**Figure 3.**
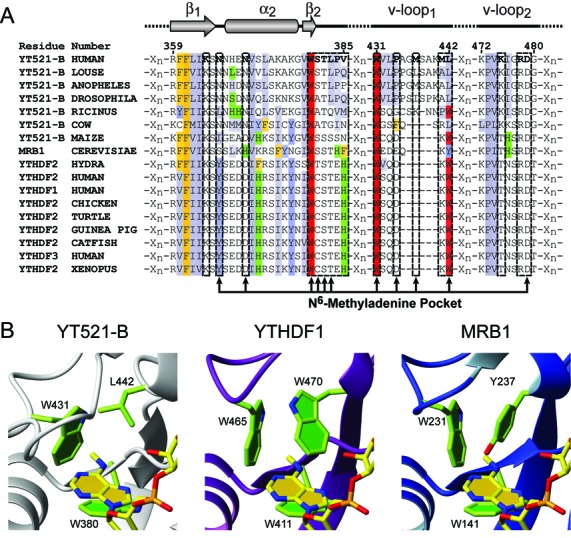
Alignment of YTH domains and homology models. (**A**) Clipped sequence alignment of a representative selection of YTH domain containing proteins. Regions shown include amino acids involved in RNA binding. Hydrophobic residues are colored gray, aromatic residues (F,Y,H,W) yellow, blue, green and red, respectively. Top: secondary structure representation of selected regions; first β-strand (β1), second α-helix (α2), second β-strand (β2) and the two variable loops (v-loop1 and v-loop2). Position of selected residues involved in m^6^A recognition are marked with arrows. (**B**) Homology models of the YTH domains of *Homo sapiens* YTHDF1 and *S. cerevisiae* MRB1 binding m^6^A. The corresponding perspective from the presented structure is shown for comparison. Representation as in (Figure 2A and C), except that the protein ribbon of YTHDF1 is in purple and the one of MRB1 in blue.

Sequence alignment of the YTH domain of different proteins from different organisms (from human to yeast) reveals that most of the residues interacting with the RNA are conserved (Figure 3A and Supplementary Figure S3). For example, Trp 380 and Trp 431 that interact with the methyl group of m^6^A are strictly conserved and Thr 382 that interacts with A3 N7 is either a Thr or a Ser. Moreover, two of the four positive side-chains (R or K) that interact with the phosphate oxygens are strictly conserved among YTH domains (Lys 364 and Arg 478) suggesting that most YTH domains could recognize specifically m^6^A-containing RNA and bind in a very similar manner as YT521-B. Based on our structure, we built homology models of the human YTHDF1 YTH domain and the *Saccharomyces cerevisiae* MRB1 YTH domain which were previously shown to bind m^6^A-containing RNA (11,12). The methylated RNA can be accommodated perfectly into these models (Figure 3B). Quite interestingly, those models revealed that the hydrophobic contacts between m^6^A_3_ and Leu 442 of YT512-B are conserved although Leu 442 is substituted by a Trp (YTHDF1), which is conserved in YTHDF2 and 3 (Figure 3A and Supplementary Figure S3), or a Tyr (MRB1) residue. In these YTH domains, the methyl group of m^6^A could therefore be encaged by three aromatic side chains (Figure 3B).

## DISCUSSION

Our structure of YT521-B YTH domain in complex with RNA confirms previous predictions (16) that the YTH domain is a single-stranded RNA-binding domain that accommodates six nucleotides. Unlike the previously reported SELEX motif of YT521-B (17), the structure reveals a weak sequence-specificity for the sequence NGANNN. More importantly, we demonstrate that N6 methylation of the adenine 3 increases the binding of YT521 YTH to the RNA by a factor of 50 (dissociation constants of 5 μM and 0.1 μM for the unmethylated and methylated RNAs, respectively) (Figure 1C). This finding is consistent with an increase in binding affinity observed for other YTH-containing protein such as YTHDF1 (20-fold), YTHDF2 (16-fold) and YTHDF3 (5-fold) (12). Our structure reveals how such a dramatic increase in affinity is achieved: the YTH domain of YT521-B contains a preformed binding pocket for the methyl group consisting of two tryptophan side-chains and one leucine. One difference between the free and the complex structure is that the loop region containing Pro 434 and Met 437 is positioned closer to the methylated adenine-binding pocket in the bound state (Supplementary Figure S2C). Based on homology models, we can predict that for the homologous proteins YTHDF1–3 and MRB1, the methyl pocket would consist of a cage of three aromatic side chains (Figure 3B). During the preparation of this manuscript a crystal structure of the YTH domain of the *Zygosaccharomyces rouxii* MRB1 protein was published, which revealed a very similar mode of recognition of m^6^A (38). This study speculates that a number of YTH domains (where one aromatic residue is substituted by a leucine) like the one presented here would not bind m^6^A. The data presented here show that the affinities measured by ITC for these domains for m^6^A containing RNA are nearly identical (0.1 μM YT521-B YTH domain and 0.2 μM MRB1 YTH domain (38)) demonstrating that the substitution of the aromatic residue by a leucine does not lead to a significant affinity change. A manuscript was published during the peer review process of this manuscript presenting the structure of the YTH domain of a homologous human protein in complex with m6A containing RNA, which reports similar findings (39)

The involvement of aromatic cages for the recognition of buried methyl groups with a cavity insertion mode has been a current theme in structural studies of methylated nucleotides (40) but also of methylated amino acids like in histone tails by their respective reader domains (41). Recognition by the YTH reader domain of m^6^A are not exception to this theme despite the fact that the m^6^A modification does not create a positive charge unlike m^7^G for example (40).

It was quite unexpected to find that the YTH domain of YT521-B has a sequence-specificity for GA-containing RNA (even without adenine methylation). Although this affinity is modest (*K*_d_ = 5 μM), this finding could have a major biological implication. It was recently found that YTH domain-containing proteins are found in the multi-subunit protein complexes responsible for m^6^A RNA modification (42). The specificity for GA could imply that the YTH domains found in these complexes might have a role in finding the RNA target since most m^6^A sites contain a GA sequence.

In conclusion the work presented here describes the first structure of a mammalian YTH domain bound to RNA and explains at the molecular level how this domain specifically recognizes m^6^A using a hydrophobic pocket composed of two to three conserved aromatic side chains. These structural data strongly support the proposal that the YTH domain functions as a reader domain for N6-methylated adenine. The high affinity and sequence-specificity of this reader domain for G-m^6^A-containing RNA further strengthen the notion that besides sequence and secondary structure, RNA modification can have a decisive influence on post-transcriptional gene regulation events.

## ACCESSION NUMBERS

Structural coordinates were deposited in the PDB database (www.pdb.org) with accession code 2MTV. Chemical shifts and restraints used for structure calculation were deposited in the Biological Magnetic Resonance Data Bank (www.bmrb.wisc.edu) with accession number 25188.

## SUPPLEMENTARY DATA

Supplementary Data are available at NAR Online.

SUPPLEMENTARY DATA
