# Supplementary material for: Solution structure of the YTH domain in complex with N6-methyladenosine RNA: a reader of methylated RNA
Source: Nucleic Acids Res. 2014 Nov 11;42(22):13911–9. doi: 10.1093/nar/gku1116 (PMC4267619; doi:10.1093/nar/gku1116)
Supplement: SUPPLEMENTARY DATA [file supp_42_22_13911__index.html]

Solution structure of the YTH domain in complex with N6-methyladenosine RNA: a reader of methylated RNA — Solution structure of the YTH domain in complex with N6-methyladenosine RNA: a reader of methylated RNA — Solution structure of the YTH domain in complex with N6-methyladenosine RNA: a reader of methylated RNA — SUPPLEMENTARY DATA 

# Solution structure of the YTH domain in complex with N6-methyladenosine RNA: a reader of methylated RNA

## SUPPLEMENTARY DATA

**Files in this Data Supplement:**

- SUPPLEMENTARY DATA
