# Supplementary material for: Solution structure of the YTH domain in complex with N6-methyladenosine RNA: a reader of methylated RNA
Source: Nucleic Acids Res. 2014 Nov 11;42(22):13911–9. doi: 10.1093/nar/gku1116 (PMC4267619; doi:10.1093/nar/gku1116)
Supplement: SUPPLEMENTARY DATA [file supp_gku1116_nar-02765-r-2014-File006.pdf]

Supplementary Information:

Solution structure of the YTH domain in complex with N^6-methyladenosine RNA: a reader of methylated RNA

Dominik Theler[1], Cyril Dominguez[1, 2] , Markus Blatter[1], Julien Boudet[1] and Frédéric H-T Allain[1,*]

[1] Institute of Molecular Biology and Biophysics, Eidgenössische Technische Hochschule (ETH) Zurich, 8093 Zurich, Switzerland

[2] Present address: Department of Biochemistry, Henry Wellcome Laboratories of Structural Biology, University of Leicester, LE1 9HN Leicester, UK

[*] Correspondence: allain@mol.biol.ethz.ch

Supplementary Results:

Supplementary Figure 1: Modified SIA approach, ITC thermodynamic parameters and NMR titration ` spectra.

Supplementary Figure 2: Structural comparison of the free and bound state of the domain and intermolecular NOEs to the N6 methyl group.

Supplementary Figure 3: Full alignment and UniProt Entries

Supplementary Figure1: Modified SIA approach, ITC Thermodynamic Parameters and NMR titration spectra

A

AGACAC 0.83	UAACNC 0.57	**UNACNC 1.00**	UNNANC 0.83	**UGACAC 0.99**	**UGACAA 0.95**
CGACAC 0.76	UCACNC 0.56	UNCCNC 0.17	**UNNCNC 1.00**	**UGACCC 0.95**	**UGACAC 1.00**
**GGACAC 1.00**	**UGACNC 1.00**	UNGCNC 0.16	UNNGNC 0.81	UGACGC 0.86	**UGACAG 0.95**
**UGACAC 0.90**	UUACNC 0.43	UNUCNC 0.37	UNNUNC 0.84	**UGACUC 1.00**	**UGACAU 0.96**
Position 1	Position 2	Position 3	Position 4	Position 5	Position 6

B

Ligand (Syringe)	Receptor (Cell)	Kd (µM)	ΔH ($kcal.mol^{-1}$)	ΔS ($cal.mol^{-1}.deg^{-1}$)	N value
**5'-UGACAC-3'**	YTH	5.35 ± 0.18	-2.23 ± 0.37	15.6	1.08 ± 0.01
**5'-UGm^{6}ACAC-3'**	YTH	0.103 ± 0.003	-14.4 ± 0.4	-17.2	1.02 ± 0.01

C

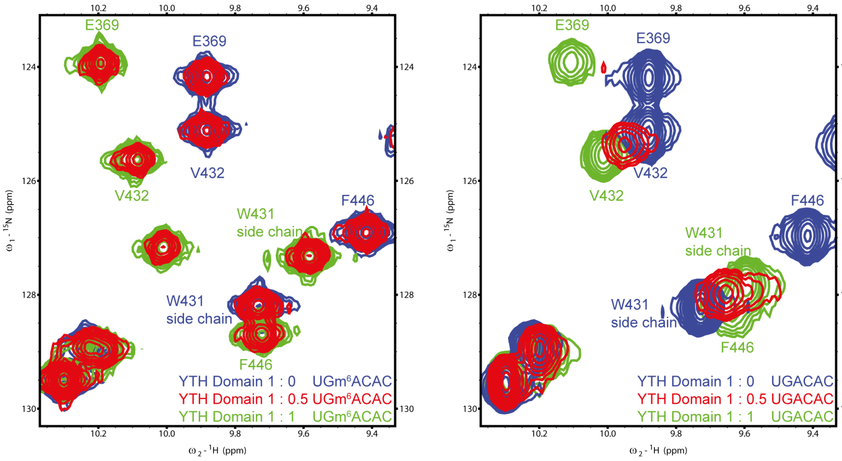

A) Results of the modified SIA approach (32) (Supplemental experimental procedures). 24 oligonucleotide sequences are tested for binding to the YTH domain. N indicates that the oligonucleotide is synthesized with a phosphoramidite pool containing all four bases equally populated. For each position, the oligonucleotides used and the obtained scores are shown. The chemical shift difference is normalized to the chemical shift difference of the oligonucleotide showing the largest amplitude. Note that position 4 was assayed first, followed by position 3, 2, 5, 6 and 1. For each position, the oligonucleotides with scores higher than 0.95 are marked in bold. Note that except for position 2 and 3 that show a clear preference for G and A, respectively, all other positions show only a weak sequence-selectivity in agreement with the structure of the complex. Individual nucleotides at the position assessed are colored. Individual oligonucleotides as well as the positions of the hexanucleotide are written left to right in the 5' to 3' direction.

B) Thermodynamic parameters (*K*, ΔH, ΔS and N) with respective errors from the ITC runs.

C) Overlay of a selected region of ^{1}H- ^{15}N HSQC spectra of titrations of the YTH domain of YT521-B with 5'-UGm^{6}ACAC-3' (left) and 5'-UGACAC-3' (right). The molar protein to RNA ratios of the titrations points are indicated. Both titrations were recorded at a magnetic field strength of 500 MHz.

Supplementary Figure 2: Structural comparison of the free and bound state of the domain and Intermolecular NOEs to the N6 methyl group.

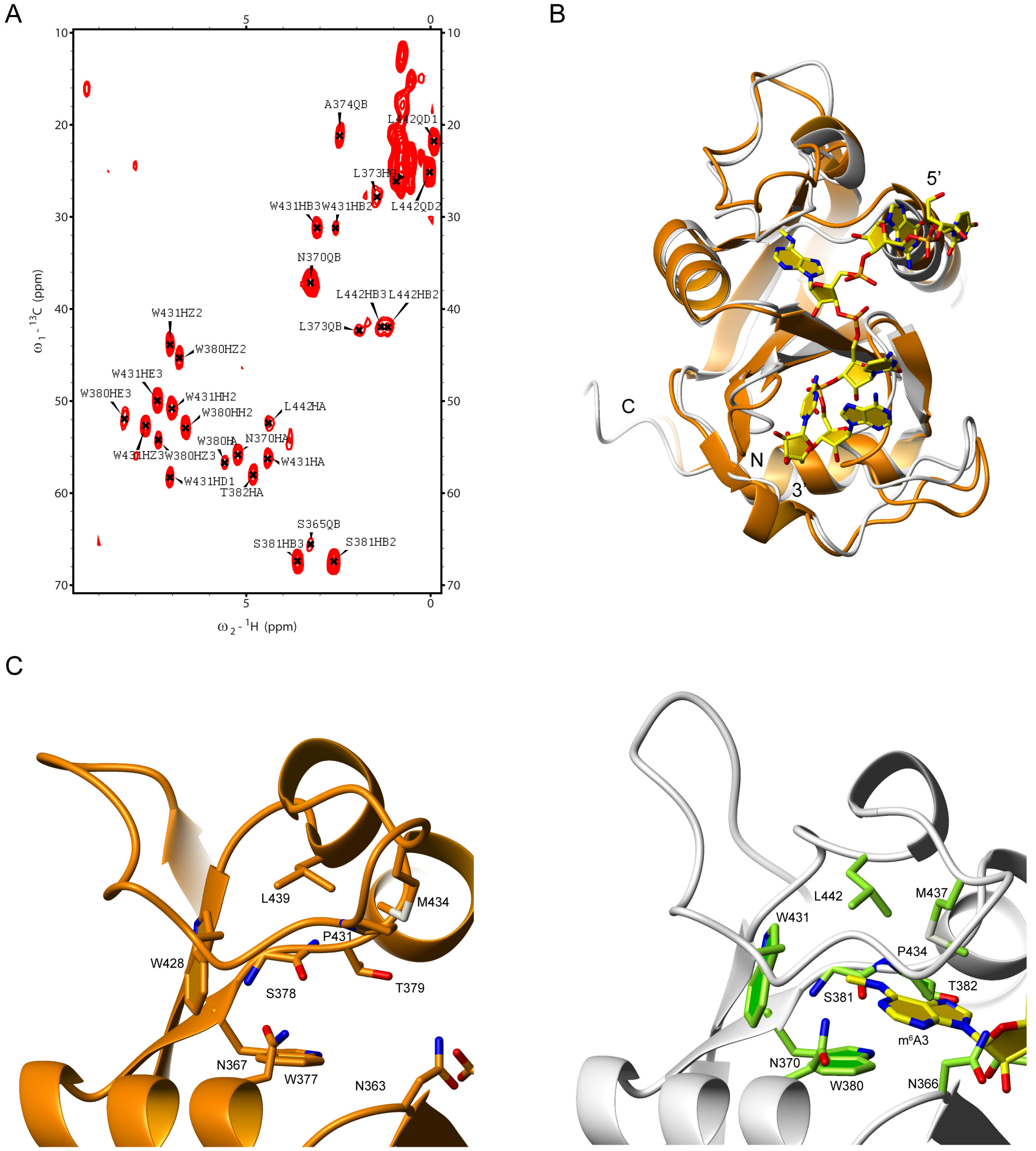

A) 2D ^{1}H-^{13}C HSQC plane (ω_3 - ^{1}H = 0.68 ppm) of the 3D ^{13}C F1-edited F3-filtered NOESY ^{1}H-^{13}C HSQC, which contains the intermolecular NOEs observed to the N6 methyl in this spectrum. Peaks are labeled with the protein proton assignment (ω_2). The intermolecular NOE to W341 HH2 was not included in the structure calculation as a restraint. This intermolecular NOE is in agreement with the solved structure.

B) Structural comparison of the YT521–B 5'-UGm^{6}ACAC-3' complex structure with the solution structure of a homologous human YTH domain (PDB ID 2YUD, YTHDC1) displayed as an orange ribbon.

C) Structural comparison of the methyl binding pocket in the free (left) and bound state (right). Coloring as in B) and Figure 2. Carbon atoms of the free domain are displayed in orange.

Figure S3: Full Alignment and UniProt Entries

A

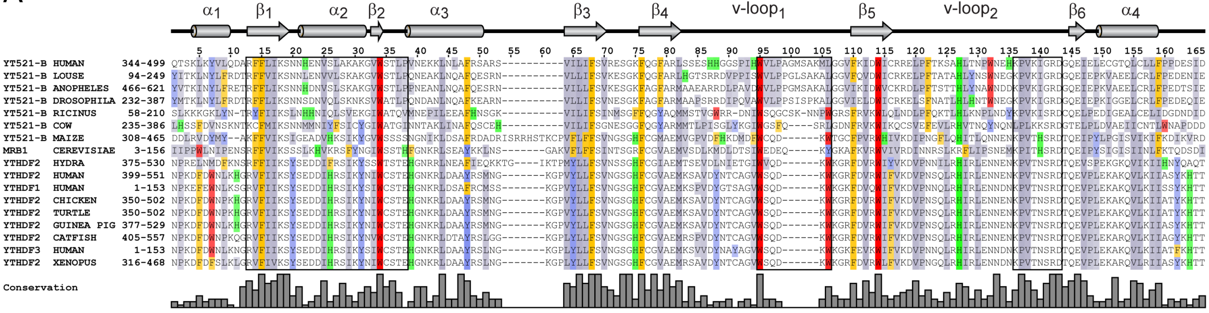

B

Alignment Name	UNIPROT Entry
YT521-B HUMAN	Q96MU7
YT521-B LOUSE	E0VM95
YT521-B ANOPHELES	W5JNR3
YT521-B DROSOPHILA	Q8IRD2
YT521-B RICINUS	B9RBY7
YT521-B COW	A7ANQ1
YT521-B MAIZE	B6SXK7
MRB1 CEREVISIAE	Q06390
YTHDF2 HYDRA	T2M255
YTHDF2 HUMAN	Q9Y5A9
YTHDF1 HUMAN	Q9BYJ9
YTHDF2 CHICKEN	F1P3X6
YTHDF2 TURTLE	K7GEE8
YTHDF2 GUINEA PIG	H0W1K7
YTHDF2 CATFISH	W5UBT8
YTHDF3 HUMAN	Q7Z739
YTHDF2 XENOPUS	Q6DCK2

A) Sequence alignment of a representative selection of YTH domain containing proteins. Boxes indicate the regions shown in Figure 3A. Hydrophobic residues are colored grey, aromatic residues (F, Y, H, W) yellow, blue, green and red respectively. Top: Secondary structure representation; first to sixth β-strand, first to fourth α-helix and the two variable loops. Bottom: Conservation bars.

B) UniProt entries used in the sequence alignment.
